# Diazido­(2,2′-bipyrid­yl)dimethanol­nickel(II)

**DOI:** 10.1107/S1600536810038377

**Published:** 2010-10-02

**Authors:** Hong-Gang Li, Shao-Ying Li, Li-Jun Shao

**Affiliations:** a Clinical College, Weifang Medical University, Weifang, Shandong 261042, People’s Republic of China; b Department of Preventative Medicine, Weifang Medical University, Weifang, Shandong 261042, People’s Republic of China

## Abstract

The title complex, [Ni(N_3_)_2_(C_10_H_8_N_2_)(CH_3_OH)_2_], lies on a twofold roation axis which runs through the Ni^II^ ion and the mid-point of the bipyridine ligand. The Ni^II^ ion is coordinated in a distorted octa­hedral environment by two azide ligands in a *trans* configuration. The methanol ligands are in a *cis* configuration and their hy­droxy groups form intra­molecular O—H⋯(N,N) hydrogen bonds with the azide ligands.

## Related literature

For related structures, see: Urtiaga *et al.* (1995 ▶); Phatchimkun *et al.* (2009 ▶); Kou *et al.* (2008 ▶); Hou (2008 ▶).
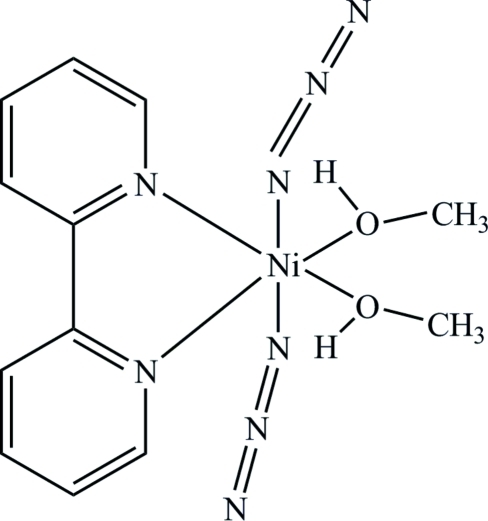


## Experimental

### 

#### Crystal data

[Ni(N_3_)_2_(C_10_H_8_N_2_)(CH_4_O)_2_]
*M* *_r_* = 363.04
Monoclinic, 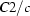

*a* = 16.8173 (15) Å
*b* = 13.4470 (12) Å
*c* = 7.1848 (6) Å
β = 111.238 (1)°
*V* = 1514.4 (2) Å^3^
*Z* = 4
Mo *K*α radiation
μ = 1.31 mm^−1^
*T* = 293 K
0.32 × 0.24 × 0.19 mm

#### Data collection

Bruker APEXII CCD diffractometer
Absorption correction: multi-scan (*SADABS*; Sheldrick, 2008*a* ▶) *T*_min_ = 0.680, *T*_max_ = 0.790
3647 measured reflections
1333 independent reflections
1247 reflections with *I* > 2σ(*I*)
*R*_int_ = 0.012

#### Refinement

*R*[*F* ^2^ > 2σ(*F* ^2^)] = 0.058
*wR*(*F* ^2^) = 0.210
*S* = 1.02
1333 reflections
106 parameters
H-atom parameters constrained
Δρ_max_ = 1.08 e Å^−3^
Δρ_min_ = −0.68 e Å^−3^

### 

Data collection: *APEX2* (Bruker, 2004 ▶); cell refinement: *SAINT-Plus* (Bruker, 2001 ▶); data reduction: *SAINT-Plus*; program(s) used to solve structure: *SHELXS97* (Sheldrick, 2008*b* ▶); program(s) used to refine structure: *SHELXL97* (Sheldrick, 2008*b* ▶); molecular graphics: *SHELXTL* (Sheldrick, 2008*b* ▶); software used to prepare material for publication: *SHELXTL*.

## Supplementary Material

Crystal structure: contains datablocks I, global. DOI: 10.1107/S1600536810038377/lh5134sup1.cif

Structure factors: contains datablocks I. DOI: 10.1107/S1600536810038377/lh5134Isup2.hkl

Additional supplementary materials:  crystallographic information; 3D view; checkCIF report

## Figures and Tables

**Table 1:** Hydrogen-bond geometry (Å, °)

*D*—H⋯*A*	*D*—H	H⋯*A*	*D*⋯*A*	*D*—H⋯*A*
O1—H1a⋯N3	0.93	2.25	2.721 (4)	111
O1—H1a⋯N4	0.93	2.48	3.227 (5)	137

**Fig. 1.**
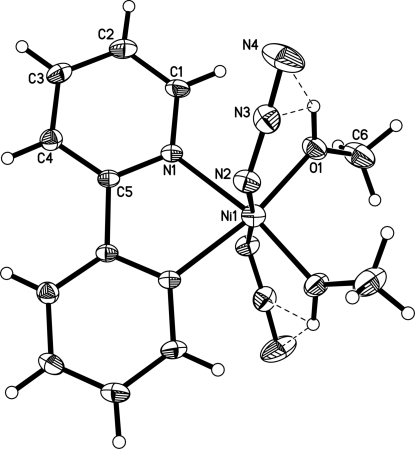
The molecular structure of the title compound with the atom-labelling scheme. Displacement ellipsoids are drawn at the 30% probability level. Hydrogen bonds are shown as dashed lines. Unlabeled atoms are related by the symmetry operator -x+2, y, -z+1/2.

## supplementary crystallographic information

### Comment

As has been known for some time, 2,2'-bipyridine is a good bidentate chelating ligand and the azido group a good bridging ligand. Here, we present a new six-coordinate nickel(II) complex based on 2,2'-bipyridine azido ligands.

The molecular structure of the title compound is shown in Fig. 1. The coordination environment of the Ni^II^ ion distorted octahedral, in which two sites are occupied by the two N atoms of the chelating 2,2'-bipyridine ligand and the *trans* positions are occupied by two N atoms of two azido ligands. Two O atoms of two methanol ligands complete the coordination. The Ni—N_bipyridine_ and Ni—N_azido_ bond lengths are basically consistant with the corresponding distances found in other nickel bipyridine azido copmplexes (Urtiaga, *et al.*, 1995; Phatchimkun, *et al.*, 2009; Kou, *et al.*, 2008; Hou, 2008.).

### Experimental

A mixture of 2,2'-bipyridine, NiCl_2_.6H_2_O (1:1, molar ratio), excessive NaN_3_ and methanol (20 ml) was sealed in a Teflon-lined autoclave (25 ml) and heated 353 K for 12 h. Upon cooling slowly and opening the bomb, green crystals suitable for X-ray diffraction were obtained with a yield about 55% (based on 2,2'-bipyridine).

### Refinement

The H atoms bonded to C atoms were placed using the HFIX commands in *SHELXL-97* (Sheldrick, 2008*a*), with C—H distances of 0.93 and 0.96 Å, and were allowed for as riding atoms with *U*_iso_(H) = 1.2*U*_eq_(C) and 1.5*U*_eq_(C), respectively. The hydroxy H atom could not be found in a difference map but by including it using the HFIX 43 instruction in *SHELXL-97* there are likely acceptors for hydrogen bonds [see Table 1].

### Crystal data

**Table:**

[Ni(N_3_)_2_(C_10_H_8_N_2_)(CH_4_O)_2_]	*F*(000) = 752
*M**_r_* = 363.04	*D*_x_ = 1.592 Mg m^−^^3^
Monoclinic, *C*2/*c*	Mo *K*α radiation, λ = 0.71073 Å
Hall symbol: -C 2yc	Cell parameters from 1564 reflections
*a* = 16.8173 (15) Å	θ = 2.8–27.4°
*b* = 13.4470 (12) Å	µ = 1.31 mm^−^^1^
*c* = 7.1848 (6) Å	*T* = 293 K
β = 111.238 (1)°	Block, green
*V* = 1514.4 (2) Å^3^	0.32 × 0.24 × 0.19 mm
*Z* = 4	

### Data collection

**Table:**

Bruker APEXII CCD diffractometer	1333 independent reflections
Radiation source: fine-focus sealed tube	1247 reflections with *I* > 2σ(*I*)
graphite	*R*_int_ = 0.012
φ and ω scans	θ_max_ = 25.0°, θ_min_ = 2.0°
Absorption correction: multi-scan (*SADABS*; Sheldrick, 2008*a*)	*h* = −15→20
*T*_min_ = 0.680, *T*_max_ = 0.790	*k* = −15→11
3647 measured reflections	*l* = −8→8

### Refinement

**Table:**

Refinement on *F*^2^	Primary atom site location: structure-invariant direct methods
Least-squares matrix: full	Secondary atom site location: difference Fourier map
*R*[*F*^2^ > 2σ(*F*^2^)] = 0.058	Hydrogen site location: inferred from neighbouring sites
*wR*(*F*^2^) = 0.210	H-atom parameters constrained
*S* = 1.02	*w* = 1/[σ^2^(*F*_o_^2^) + (0.1511*P*)^2^ + 7.4217*P*] where *P* = (*F*_o_^2^ + 2*F*_c_^2^)/3
1333 reflections	(Δ/σ)_max_ = 0.011
106 parameters	Δρ_max_ = 1.08 e Å^−^^3^
0 restraints	Δρ_min_ = −0.68 e Å^−^^3^

### Special details

**Table:**

Geometry. All e.s.d.'s (except the e.s.d. in the dihedral angle between two l.s. planes) are estimated using the full covariance matrix. The cell e.s.d.'s are taken into account individually in the estimation of e.s.d.'s in distances, angles and torsion angles; correlations between e.s.d.'s in cell parameters are only used when they are defined by crystal symmetry. An approximate (isotropic) treatment of cell e.s.d.'s is used for estimating e.s.d.'s involving l.s. planes.
Refinement. Refinement of *F*^2^ against ALL reflections. The weighted *R*-factor *wR* and goodness of fit *S* are based on *F*^2^, conventional *R*-factors *R* are based on *F*, with *F* set to zero for negative *F*^2^. The threshold expression of *F*^2^ > σ(*F*^2^) is used only for calculating *R*-factors(gt) *etc*. and is not relevant to the choice of reflections for refinement. *R*-factors based on *F*^2^ are statistically about twice as large as those based on *F*, and *R*- factors based on ALL data will be even larger.

### Fractional atomic coordinates and isotropic or equivalent isotropic displacement parameters (Å^2^)

**Table:**

	*x*	*y*	*z*	*U*_iso_*/*U*_eq_	
Ni1	1.0000	0.74532 (6)	0.2500	0.0357 (4)	
O1	0.9127 (2)	0.6557 (3)	0.1994 (5)	0.0403 (8)	
H1A	0.8679	0.6589	0.0766	0.048*	
N1	0.9185 (2)	0.8653 (3)	0.2091 (5)	0.0267 (8)	
N2	0.9797 (3)	0.7545 (3)	−0.0390 (6)	0.0331 (10)	
N3	0.9118 (3)	0.7278 (3)	−0.1552 (6)	0.0393 (10)	
N4	0.8473 (3)	0.7061 (5)	−0.2714 (8)	0.0713 (16)	
C1	0.8326 (2)	0.8578 (3)	0.1560 (6)	0.0347 (10)	
H1	0.8083	0.7953	0.1514	0.042*	
C2	0.7806 (3)	0.9397 (4)	0.1091 (7)	0.0397 (11)	
H2	0.7220	0.9327	0.0750	0.048*	
C3	0.8157 (3)	1.0328 (4)	0.1126 (6)	0.0374 (10)	
H3	0.7811	1.0889	0.0782	0.045*	
C4	0.9035 (3)	1.0414 (3)	0.1684 (6)	0.0323 (9)	
H4	0.9287	1.1033	0.1723	0.039*	
C5	0.9526 (2)	0.9565 (3)	0.2179 (5)	0.0244 (8)	
C6	0.9097 (4)	0.5858 (5)	0.3297 (8)	0.0599 (16)	
H6A	0.9663	0.5619	0.4021	0.090*	
H6B	0.8745	0.5317	0.2589	0.090*	
H6C	0.8863	0.6138	0.4215	0.090*	

### Atomic displacement parameters (Å^2^)

**Table:**

	*U*^11^	*U*^22^	*U*^33^	*U*^12^	*U*^13^	*U*^23^
Ni1	0.0333 (6)	0.0382 (6)	0.0347 (6)	0.000	0.0114 (4)	0.000
O1	0.0367 (17)	0.0404 (19)	0.0380 (17)	−0.0107 (14)	0.0068 (13)	0.0056 (14)
N1	0.0201 (16)	0.0322 (18)	0.0275 (17)	−0.0002 (13)	0.0083 (13)	0.0000 (13)
N2	0.032 (2)	0.043 (2)	0.024 (2)	−0.0043 (14)	0.0095 (18)	0.0006 (13)
N3	0.048 (3)	0.041 (2)	0.031 (2)	−0.0041 (19)	0.016 (2)	0.0055 (17)
N4	0.057 (3)	0.100 (4)	0.042 (3)	−0.034 (3)	0.000 (2)	0.003 (3)
C1	0.0205 (19)	0.043 (3)	0.038 (2)	−0.0039 (17)	0.0078 (16)	0.0002 (18)
C2	0.024 (2)	0.054 (3)	0.041 (2)	0.0030 (19)	0.0108 (18)	−0.002 (2)
C3	0.029 (2)	0.043 (3)	0.037 (2)	0.0113 (18)	0.0087 (18)	0.0006 (18)
C4	0.031 (2)	0.033 (2)	0.032 (2)	0.0023 (17)	0.0103 (17)	−0.0023 (17)
C5	0.021 (2)	0.033 (2)	0.0198 (17)	0.0004 (15)	0.0069 (14)	−0.0010 (15)
C6	0.052 (3)	0.069 (4)	0.049 (3)	−0.024 (3)	0.008 (2)	0.008 (3)

### Geometric parameters (Å, °)

**Table:**

Ni1—O1	1.831 (3)	C1—C2	1.370 (7)
Ni1—O1^i^	1.831 (3)	C1—H1	0.9300
Ni1—N2	1.982 (4)	C2—C3	1.380 (7)
Ni1—N2^i^	1.982 (4)	C2—H2	0.9300
Ni1—N1^i^	2.068 (4)	C3—C4	1.387 (6)
Ni1—N1	2.068 (4)	C3—H3	0.9300
O1—C6	1.340 (6)	C4—C5	1.379 (6)
O1—H1A	0.9300	C4—H4	0.9300
N1—C5	1.346 (5)	C5—C5^i^	1.490 (7)
N1—C1	1.358 (5)	C6—H6A	0.9600
N2—N3	1.199 (6)	C6—H6B	0.9600
N3—N4	1.142 (6)	C6—H6C	0.9600
			
O1—Ni1—O1^i^	97.7 (2)	N4—N3—N2	176.8 (5)
O1—Ni1—N2	90.64 (15)	N1—C1—C2	121.9 (4)
O1^i^—Ni1—N2	94.04 (16)	N1—C1—H1	119.1
O1—Ni1—N2^i^	94.04 (16)	C2—C1—H1	119.1
O1^i^—Ni1—N2^i^	90.64 (15)	C1—C2—C3	119.6 (4)
N2—Ni1—N2^i^	172.9 (2)	C1—C2—H2	120.2
O1—Ni1—N1^i^	169.78 (14)	C3—C2—H2	120.2
O1^i^—Ni1—N1^i^	92.41 (16)	C2—C3—C4	119.0 (4)
N2—Ni1—N1^i^	87.38 (14)	C2—C3—H3	120.5
N2^i^—Ni1—N1^i^	87.08 (15)	C4—C3—H3	120.5
O1—Ni1—N1	92.41 (16)	C5—C4—C3	118.8 (4)
O1^i^—Ni1—N1	169.78 (14)	C5—C4—H4	120.6
N2—Ni1—N1	87.08 (15)	C3—C4—H4	120.6
N2^i^—Ni1—N1	87.38 (14)	N1—C5—C4	122.5 (3)
N1^i^—Ni1—N1	77.48 (19)	N1—C5—C5^i^	113.9 (2)
C6—O1—Ni1	123.5 (3)	C4—C5—C5^i^	123.6 (2)
C6—O1—H1A	118.2	O1—C6—H6A	109.5
Ni1—O1—H1A	118.2	O1—C6—H6B	109.5
C5—N1—C1	118.2 (3)	H6A—C6—H6B	109.5
C5—N1—Ni1	117.0 (2)	O1—C6—H6C	109.5
C1—N1—Ni1	124.4 (3)	H6A—C6—H6C	109.5
N3—N2—Ni1	118.1 (3)	H6B—C6—H6C	109.5

Symmetry codes: (i) −*x*+2, *y*, −*z*+1/2.

### Hydrogen-bond geometry (Å, °)

**Table:**

*D*—H···*A*	*D*—H	H···*A*	*D*···*A*	*D*—H···*A*
O1—H1a···N3	0.93	2.25	2.721 (4)	111
O1—H1a···N4	0.93	2.48	3.227 (5)	137

## References

[bb1] Bruker (2001). *SAINT-Plus* Bruker AXS Inc., Madison,Wisconsin, USA.

[bb2] Bruker (2004). *APEX2* Bruker AXS Inc., Madison, Wisconsin, USA.

[bb3] Hou, J. (2008). *Acta Cryst.* E**64**, m1571. 10.1107/S1600536808037902 PMC2959945 21581175

[bb4] Kou, H. Z., Hishiya, S. & Sato, O. (2008). *Inorg. Chim. Acta*, **361**, 2396–2406.

[bb5] Phatchimkun, J., Kongsaeree, P., Suchaichit, N. & Chaichit, N. (2009). *Acta Cryst.* E**65**, m1020–m1021. 10.1107/S1600536809029407 PMC2970126 21577389

[bb6] Sheldrick, G. M. (2008*a*). *SADABS* University of Göttingen, Germany.

[bb7] Sheldrick, G. M. (2008*b*). *Acta Cryst.* A**64**, 112–122. 10.1107/S0108767307043930 18156677

[bb8] Urtiaga, M. K., Arriortua, M. I., De Muro, I. G. & Cortes, R. (1995). *Acta Cryst.* C**51**, 62–65.

